# DNA–histone cross-link locks the nucleosome structure and disrupts its recognition and processing

**DOI:** 10.1093/procel/pwaf094

**Published:** 2025-11-05

**Authors:** Xiajing Shan, Gaoyuan Ji, Jiahui Li, Mengtian Ren, Jingke Ma, Yifei Zhou, Haitao Li, Chuanzheng Zhou

**Affiliations:** State Key Laboratory of Elemento-Organic Chemistry, Frontiers Science Center for New Organic Matter, Department of Chemical Biology, College of Chemistry, Nankai University, Tianjin 300071, China; MOE Key Laboratory of Protein Sciences, Beijing Frontier Research Center for Biological Structure, Beijing Advanced Innovation Center for Structural Biology, Department of Basic Medical Sciences, School of Medicine, Tsinghua University, Beijing 100084, China; State Key Laboratory of Elemento-Organic Chemistry, Frontiers Science Center for New Organic Matter, Department of Chemical Biology, College of Chemistry, Nankai University, Tianjin 300071, China; State Key Laboratory of Elemento-Organic Chemistry, Frontiers Science Center for New Organic Matter, Department of Chemical Biology, College of Chemistry, Nankai University, Tianjin 300071, China; State Key Laboratory of Elemento-Organic Chemistry, Frontiers Science Center for New Organic Matter, Department of Chemical Biology, College of Chemistry, Nankai University, Tianjin 300071, China; State Key Laboratory of Elemento-Organic Chemistry, Frontiers Science Center for New Organic Matter, Department of Chemical Biology, College of Chemistry, Nankai University, Tianjin 300071, China; MOE Key Laboratory of Protein Sciences, Beijing Frontier Research Center for Biological Structure, Beijing Advanced Innovation Center for Structural Biology, Department of Basic Medical Sciences, School of Medicine, Tsinghua University, Beijing 100084, China; State Key Laboratory of Elemento-Organic Chemistry, Frontiers Science Center for New Organic Matter, Department of Chemical Biology, College of Chemistry, Nankai University, Tianjin 300071, China; Haihe Laboratory of Sustainable Chemical Transformations, Tianjin 300192, China; State Key Laboratory of Medicinal Chemical Biology, Nankai University, Tianjin 300353, China

**Dear Editor,**

DNA-protein cross-links (DPCs) are continuously formed within cells, either as a result of DNA damage (Tretyakova et al., 2015) or as intermediates during the endogenous DNA metabolism (Weickert and Stingele, 2022). The formation of DPCs can severely disrupt the essential functions of DNA (Pujari et al., 2021). Recently, we and others have demonstrated that DNA damage and repair in nucleosomes are accompanied by the formation of DNA–histone cross-links (DHCs) (Ren et al., 2022). DHC can be regarded as both DNA modifications and histone modifications. The covalent cross-link of DNA and histones in nucleosomes is believed to significantly alter the structural dynamics of the nucleosome (Frouws et al., 2018), which, in turn, may profoundly affect DNA function. Unfortunately, the regulation of nucleosome structure and function through DHC formation has been rarely explored, primarily because the naturally occurring DHCs are either unstable or formed in very low yields. In the present study, we prepared nucleosomes with stable DHCs in a site-specific manner, allowing us to systematically evaluate how DHCs regulate the structure and function of nucleosomes.

The effect of DHCs on nucleosome structure and function was investigated using reconstituted nucleosome core particles (NCPs) containing the “601” DNA sequence and human histones. The “601” DNA sequence is known to wrap around the human histone octamer to form a stable and well-positioned NCP, whose crystal structure has been resolved (Vasudevan et al., 2010). Site-specific DHC was introduced at the dyad region of the NCP via a copper-­catalyzed azide–alkyne cycloaddition (CuAAC) reaction (Fig. 1A). Specifically, we generated a human H3 mutant, H3-C96S-C110S-K115C, in which the native Cys residues at position 96 and 110 were replaced with serine, and K115 was mutated to Cys. This histone mutant contains only a single Cys residue at position 115. Treatment of this mutant with azido-PEG_4_-iodoacetamide (IA-PEG_4_-N_3_) yielded azido-modified H3 (Fig. S1). Meanwhile, we synthesized an oligonucleotide corresponding to the 66–82 fragment of “601” DNA via solid-phase DNA synthesis (Table S1). In this sequence, dT74 was replaced with 5-(1,7-octadiynyl)-5′-deoxyuridine (octadiynyl-dU, Fig. S1). Following enzymatic ligation and annealing with the complementary DNA strand, we successfully obtained “601” DNA containing an alkynyl-dU modification at position 74. The modified strand also carried a 5′-FAM (or 5′-Cy5) fluorophore to facilitate subsequent analyses (Fig. S2).

**Figure 1.**
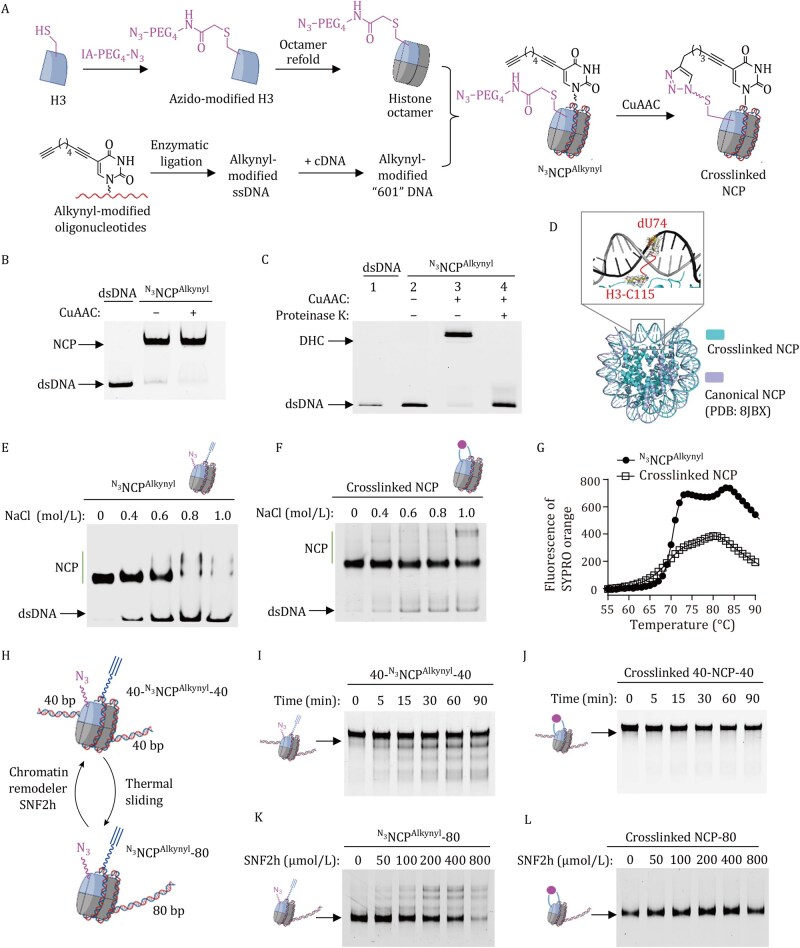
**Effect of DNA–histone cross-link (DHC) on nucleosome structure and dynamics**. (A) Diagram illustrating the strategy for preparing NCPs with site-specific DHCs. CuAAC, copper-catalyzed azide–alkyne cycloaddition reaction. (B) 5% (*w*/*v*) native PAGE analysis demonstrating the structural integrity of nucleosomes. (C) 10% SDS-PAGE analysis showing the formation of DHCs in NCPs through click chemistry. Gels were visualized by fluorescence imaging of FAM-labeled DNA. (D) Structural superimposition of the cryo-EM structure of cross-linked NCP and the canonical NCP (PDB ID: 8JBX). (E and F) EMSA analysis of the structural stability of uncrosslinked ^N3^NCP^Alkynyl^ and cross-linked NCPs under high-salt conditions. Gels were visualized by fluorescence imaging of FAM-labeled DNA. (G) Fluorescence analysis of the temperature-dependent disassembly of crosslinked and uncrosslinked NCPs, using SYPRO Orange. The fluorescence intensity of SYPRO Orange is proportional to the amount of free histones released from the NCP, as the dye binds to free histones and becomes fluorescent upon binding. (H) Diagram illustrating the structure and sliding of centrally positioned 40-^N3^NCP^Alkynyl^-40 and end-positioned ^N3^NCP^Alkynyl^-80. (I and J) EMSA analyses of thermally induced sliding for uncrosslinked 40-^N3^NCP^Alkynyl^-40 and cross-linked 40-NCP-40. (K and L) EMSA analyses of SNF2h-mediated active sliding for uncrosslinked ^N3^NCP^Alkynyl^-80 and cross-linked NCP-80. Gels were visualized by fluorescence imaging of FAM-labeled DNA.

The azido-modified H3 prepared above was assembled with native human histones H4, H2A, and H2B to form a histone octamer, which was then reconstituted with the alkynyl-modified “601” DNA to generate uncrosslinked NCPs (^N^^3^NCP^Alkynyl^). Subsequently, CuAAC-mediated cross-linking was initiated by adding CuSO_4_, BTTAA (2-[4-[(bis[(1-*tert*-butyl-1*H*-1,2,3-triazol-4-yl)methyl]amino)methyl]-1*H*-1,2,3-triazol-1-yl]acetic acid), and sodium ascorbate. Electrophoretic mobility shift assay (EMSA) demonstrated that the NCPs retained their structural integrity after Cu(I) treatment (Fig. 1B). Analysis using 10% SDS–PAGE revealed the absence of free DNA and the appearance of a new, slower-migrating product (lane 3, Fig. 1C). Treatment of this product with proteinase K converted it back to free DNA (lane 4, Fig. 1C), suggesting that the new product was a DHC. In contrast, no DHC was observed when NCPs were incubated without CuSO_4_, BTTAA, and sodium ascorbate (lane 2, Fig. 1C), confirming that DHC formation resulted from the clicking reaction between the azide at residue C115 of H3 and the octadiynyl modification at position dU74 of “601” DNA.

The DHC was introduced between H3-C115 and DNA-dU74 at the dyad region for the following reasons. First, at the dyad, DNA interacts extensively with histones—not only with H3 and H4 but also with H2A (Lee and Hayes, 1998). Both our previous studies and those of others have demonstrated that *in situ* generation of electrophilic DNA damage at the dyad region leads to extensive DHC formation (Guschin et al., 1998). Second, each NCP contains two copies of histone H3 but only a single DNA molecule. In the modified NCP, both azido-functionalized H3-C115 residues are positioned in close proximity to the alkynyl-modified dU74 at the dyad (Fig. S1C). Competition between the two C115-azido groups for the single dU74-alkynyl site favors the formation of a single DHC at the dyad, resulting in an exceptionally high cross-linking yield. Thus, introducing a DHC at this site via click chemistry ensures the production of cross-linked nucleosomes with high purity and homogeneity.

To investigate the effect of DHC on the static structure of the NCP, we determined the cryo-EM structure of the cross-linked NCP at 3.2 Å resolution (Fig. 1D and S4; Table S2). The overall architecture closely resembled that of the canonical human NCP (PDB: 8JBX) (Ding et al., 2024), with a root-mean-square deviation of 0.546 Å. Due to inherent flexibility, the linker region between C115 and dU74 was not resolved. Nevertheless, the electron densities corresponding to C115, dU74, and surrounding residues clearly indicated that the presence of the linker did not disrupt base pairing or DNA–histone interactions (Fig. 1D). Collectively, these findings suggest that DHC does not induce significant alterations in either the local conformation near the cross-linking site or the global structure of the NCP.

Next, we evaluated the stability of uncrosslinked ^N^^3^NCP^Alkynyl^ versus cross-linked NCP under high-salt conditions. The reconstituted NCP samples initially contained approximately 0.1 mol/L NaCl. Upon increasing the NaCl concentration to 0.4 mol/L, ∼16% of the DNA dissociated from the histone core in uncrosslinked ^N^^3^NCP^Alkynyl^ (Fig. 1E). When the NaCl concentration reached 1 mol/L, intact uncrosslinked NCPs were almost completely lost. In contrast, over 91% of the cross-linked NCPs remained intact under the same conditions (Fig. 1F). These results demonstrate that cross-linked NCPs possess significantly greater structural stability than their uncrosslinked counterparts in high-salt environments.

The thermal disassembly of NCPs was monitored using SYPRO Orange, a dye that binds to free histones and becomes fluorescent upon binding (Taguchi et al., 2014). Accordingly, SYPRO Orange was added to the prepared NCP samples, and fluorescence was recorded as a function of temperature. The uncrosslinked ^N^^3^NCP^Alkynyl^ displayed a biphasic denaturation profile (Fig. 1G). As previously reported (Taguchi et al., 2014), the first peak at 70°C corresponds to the dissociation of the H2A–H2B dimer, while the second peak at 81°C corresponds to the dissociation of the H3-H4 tetramer. In contrast, the cross-linked NCP exhibited a monophasic denaturation profile, with a single peak at 80°C and fluorescence intensity approximately half that of its uncrosslinked counterpart. These results suggest that, in the cross-linked NCP, a higher temperature is required to dissociate the H2A–H2B dimer, whereas the dissociation of the H3–H4 tetramer is completely inhibited. Taken together, these findings indicate that the formation of DHCs enhances the resistance of NCPs to destabilizing conditions such as high salt and elevated temperature. In other words, DHCs substrantially improve NCP structural stability.

To investigate how DHC affects nucleosome sliding, we assembled two types of nucleosomes using the azido-modified histone octamer and 225 bp alkynyl-modified dsDNA. The first, termed 40-^N^^3^NCP^Alkynyl^-40, contains the alkynyl-modified “601” DNA at the center, flanked by two 40 bp linker regions. The second, termed ^N^^3^NCP^Alkynyl^-80, contains the “601” DNA at one end and an 80 bp linker at the other (Fig. 1H and S2). The resulting nucleosomes were subjected to a CuAAC reaction, yielding cross-linked 40-NCP-40 and cross-linked NCP-80, respectively (Fig. S5). It is known that centrally positioned nucleosomes migrate more slowly than their end-positioned counterparts in EMSA (Bilokapic et al., 2018), making 40-^N^^3^NCP^Alkynyl^-40 and ^N^^3^NCP^Alkynyl^ -80 ideal paired samples for studying nucleosome sliding using native PAGE.

Both uncrosslinked 40-^N^^3^NCP^Alkynyl^-40 and cross-linked 40-NCP-40 were incubated at 60°C, and nucleosome sliding was monitored using 7% native PAGE. For uncrosslinked 40-^N^^3^NCP^Alkynyl^-40, a series of faster-migrating bands appeared and increased over time (Fig. 1I), indicating that thermal nucleosome sliding had occurred, resulting in heterogeneous sliding states. In contrast, cross-linked 40-NCP-40 remained unchanged even after 90 min of incubation at 60°C (Fig. 1J), suggesting that DHC completely inhibits thermal nucleosome sliding.

The ATP-dependent chromatin remodeler SNF2h actively drives controlled nucleosome repositioning through ATP hydrolysis, generally shifting end-positioned nucleosomes toward central positions. We treated end-positioned nucleosomes—uncrosslinked ^N^^3^NCP^Alkynyl^-80 and cross-linked NCP-80—with different concentrations of SNF2h for 30 min. EMSA analysis showed that SNF2h induced sliding of uncrosslinked ^N^^3^NCP^Alkynyl^-80 but not cross-linked NCP-80 (Fig. 1K and 1L). It is worth noting that SNF2h exhibited comparable binding affinities toward the uncrosslinked and cross-linked nucleosomes (Fig. S6). Thus, DHC formation within nucleosomes does not affect SNF2h binding but completely abolishes its nucleosome-sliding activity.

Next, we employed SP6 RNA polymerase—one of the most efficient enzymes for transcribing nucleosomal DNA (Bondarenko et al., 2006)—to investigate how DHC affects transcription within nucleosomes. To this end, we prepared a nucleosomal substrate, SP6-^N^^3^NCP^Alkynyl-AS^ (Fig. 2A and S3), which consists of the modified nucleosome ^N^^3^NCP^Alkynyl-AS^ linked to a DNA fragment carrying the SP6 promoter. In this construct, the alkynyl-modified dU74 is positioned on the antisense strand of the SP6 transcription template. A control construct, SP6-^N^^3^NCP^Alkynyl-SS^, was also prepared, which is identical in structure except that the alkynyl modification was introduced at dU125 on the sense strand (Fig. 2B). Both nucleosomes were subjected to CuAAC reactions to generate cross-linked SP6-nucleosome^AS^ and cross-linked SP6-nucleosome^SS^, respectively (Fig. S5). Then, transcription was initiated by incubating SP6-^N^^3^NCP^Alkynyl-SS^ with SP6 RNA polymerase and an NTP mixture. After 30 min, the transcripts were analyzed using 3′ RACE (3′ rapid amplification of cDNA ends, Fig. S7A). Briefly, transcripts were first polyadenylated using poly(A) polymerase, followed by reverse transcription with a poly(T) primer. The resulting cDNA was amplified by PCR. Agarose gel analysis of the amplified products revealed a dominant band of approximately 250 bp (lane 2, Fig. 2C). This band was gel-extracted, cloned into a blunt-end vector, and subjected to Sanger sequencing, which identified it as the run-off transcript (Fig. S7B). Transcription of SP6-^N^^3^NCP^Alkynyl-AS^ yielded a mixture of full-length and shorter transcripts (lane 4, Fig. 2C). These results indicate that neither the azide modification on histone H3 nor the alkynyl modification on DNA abolishes SP6-mediated transcription within nucleosomes. However, the presence of the alkynyl group on the antisense strand appears to negatively affect transcription efficiency.

**Figure 2.**
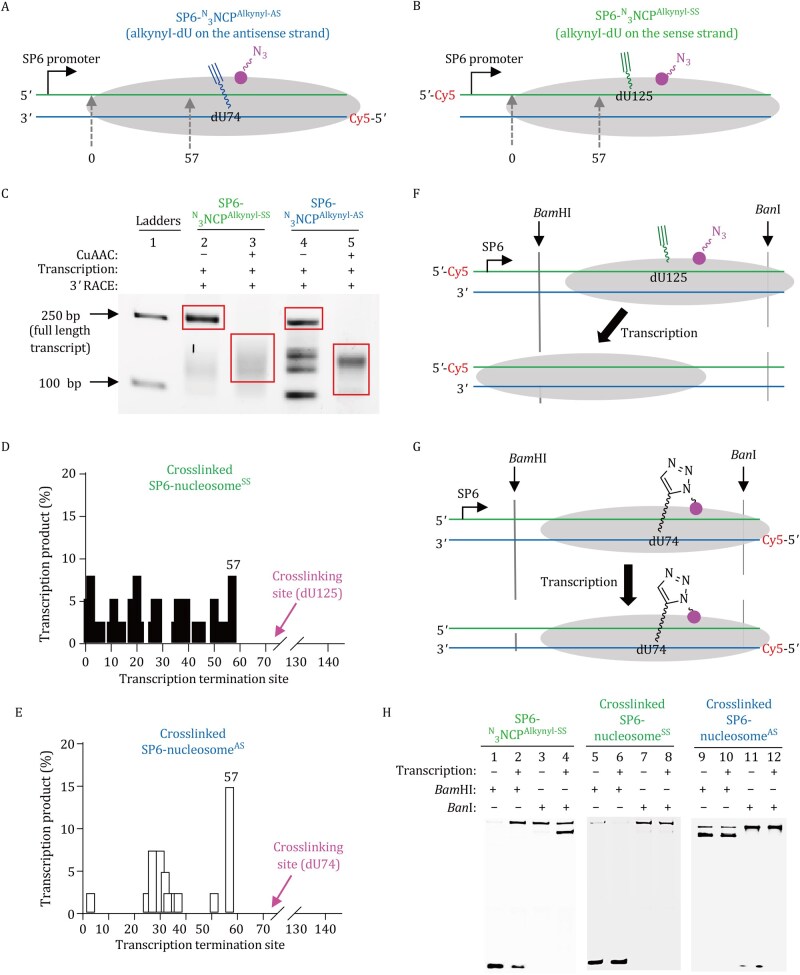
**DNA–histone cross-link blocks SP6 RNA polymerase-mediated transcription within nucleosomes**. (A and B) Nucleosome constructs used for *in vitro* transcription by SP6 RNA polymerase. (C) 3% agarose gel analysis of the amplified transcripts. The region marked with a red box was excised, purified, and used for vector cloning and Sanger sequencing. (D and E) Quantification of each transcription product plotted according to the corresponding transcription termination site. (F) Schematic representation of transcription-driven translocation and changes in restriction enzyme protection pattern for SP6-^N^^3^NCP^Alkynyl-SS^. (G) Schematic representation of the restriction enzyme protection pattern for cross-linked SP6-nucleosome^AS^. (H) Denaturing PAGE analysis showing transcription-induced changes in the restriction enzyme protection patterns across different nucleosome substrates. Gels were visualized by fluorescence imaging of Cy5-labeled DNA.

In contrast, transcription of cross-linked SP6-nucleosome^AS^ and cross-linked SP6-nucleosome^SS^ failed to produce full-length transcripts. Agarose gel analysis of the amplified transcripts revealed diffuse bands (lanes 3 and 5 in Fig. 2C). Sanger sequencing of these bands showed that they represent transcriptional pause products, with pause sites spanning positions 0 to +57 on the “601” DNA sequence (Fig. 2D and 2E). In both cases, the longest transcripts terminate at position +57, approximately 15 bp upstream of the cross-linking sites (dU74 in nucleosome^AS^ and dU125 in nucleosome^SS^). Importantly, EMSA analysis of the transcription reactions showed that both uncrosslinked SP6-^N^^3^NCP^Alkynyl^ and cross-linked nucleosomes (SP6-nucleosome^AS^ and SP6-nucleosome^SS^) remained intact after transcription (Fig. S5G). This indicates that SP6 RNA polymerase can transcribe nucleosomal DNA without disassembling the nucleosome structure. Collectively, these findings demonstrate that DHC markedly enhances the transcriptional barrier imposed by the nucleosome, causing premature stalling of SP6 RNA polymerase approximately 15 bp upstream of the cross-linking site.

It has been reported that, in free dsDNA, DNA–protein cross-links (DPCs) on the antisense strand repress transcription, whereas those on the sense strand do not (Ji et al., 2019). In such cases, steric hindrance caused by DPC appears to play a key role in transcriptional repression. However, our observation that both SP6-nucleosome^AS^ and SP6-nucleosome^SS^ lead to premature termination within nucleosomes suggests that, in this context, DHC halts transcription through a distinct mechanism.

Run-off transcription of nucleosomal DNA by SP6 RNA polymerase requires translocation of the histone octamer along the DNA (Studitsky et al., 1995). To assess whether transcription-induced nucleosome translocation occurs, we performed a restriction enzyme protection assay using ­SP6-^N^^3^NCP^Alkynyl-SS^. Before transcription, the BamHI recognition site is exposed, whereas the BanI site is occluded by the nucleosome (Fig. 2F). Digestion of SP6-^N^^3^NCP^Alkynyl-SS^ with BamHI and BanI yielded 96% cleavage at the BamHI site (lane 1, Fig. 2H) and 9% cleavage at the BanI site (lane 3, Fig. 2H). After transcription, cleavage at the BamHI site decreased to 33% (lane 2, Fig. 2H), while cleavage at the BanI site increased to 65% (lane 4, Fig. 2H), indicating that more than half of the nucleosomes had undergone translocation. This result is consistent with the presence of full-length transcripts as the major transcription products. In contrast, the restriction enzyme protection patterns of cross-linked SP6-nucleosome^AS^ and SP6-nucleosome^SS^ remained unchanged before and after transcription (Fig. 2G and 2H), indicating that no translocation occurred during transcription.

Considering that steric hindrance from a DPC represses transcription only when the cross-link is located on the antisense strand, the observation of premature transcription termination approximately 15 bp upstream of the cross-link site in both cross-linked nucleosome^AS^ and cross-linked nucleosome^SS^ suggests that steric hindrance from DHC is not the primary cause of transcriptional arrest. Instead, the absence of nucleosome translocation during transcription—an essential prerequisite for run-off transcription—in both cross-linked SP6-nucleosome^AS^ and cross-linked SP6-nucleosome^SS^ indicates that DHC-induced inhibition of nucleosome translocation is responsible for the premature termination observed in these cross-linked nucleosomes.

In recent years, the repair of DPCs has attracted considerable attention. In principle, DPCs are repaired through a two-step process: first, proteases or the proteasome recognize and selectively degrade the cross-linked proteins into peptide fragments; second, the resulting DNA–peptide conjugates are removed via classical DNA repair mechanisms, such as the nucleotide excision repair (NER) pathway (Pachva et al., 2020). To assess whether DNA-histone cross-links within nucleosomes interfere with the protease-initiated repair pathway, we treated cross-linked NCPs with proteinase K, a potent protease known to degrade histones within nucleosomes. EMSA analysis revealed that the cross-linked NCPs remained intact after 1 h of incubation (Fig. S9A). In contrast, under the same conditions, the uncrosslinked ^N^^3^NCP^Alkynyl^ was completely degraded to free dsDNA. These results indicate that DHC formation within the nucleosome renders histones more resistant to proteolytic digestion.

To further understand how DHC enhances histone resistance to proteolysis, we treated cross-linked NCPs with trypsin, an arginine- and lysine-specific protease. Analysis by 10% SDS-PAGE showed that the dsDNA-H3 cross-link within the nucleosome was degraded into a short product (fragment I), which migrated slightly faster than the intact substrate (Fig. S9B). As a control, we independently prepared a free DNA-histone cross-link (DHC) by conjugating an alkynyl-dU74-modified “601” DNA to azido-modified histone H3. Trypsin treatment of this free DHC rapidly generated fragment I. However, fragment I was further degraded into a shorter product, fragment II, which likely corresponds to “601” dsDNA tagged with a small peptide fragment. The cross-linking site on histone H3 (C115) resides in the bridge region between the *N*-terminal tail and the core domain of H3. The *N*-terminal tail, rich in lysine and arginine residues and existing as a flexible random coil, is more susceptible to trypsin digestion than the structured core domain. Accordingly, digestion of the *N*-terminal tail in both the free DHC and cross-linked NCPs rapidly yields fragment I. While the core region of H3 in the free DHC is slowly degraded over time, digestion of the core region in the cross-linked NCP is completely inhibited. These findings suggest that DHC formation within nucleosomes protects the histone core domains from protease digestion. This resistance may be attributed to the structural dynamics of the nucleosome: in uncrosslinked nucleosomes, transient DNA unwrapping and histone eviction allow proteases to access core histone regions. In contrast, DHC locks the nucleosome in a more rigid and stable conformation, as shown in our previous analyses. The loss of structural dynamics likely impedes protease binding and access to core histones. Consequently, DHCs embedded within nucleosomes are unlikely to be repaired via conventional pathways that evolved to remove free DPCs.

In conclusion, we established a site-specific DHC at the dyad of nucleosomes via a copper-catalyzed azide–alkyne cycloaddition, enabling systematic analysis of its structural and functional consequences. We demonstrate that a single DHC markedly stabilizes nucleosomes under adverse conditions—including high salt and elevated temperature—while completely abolishing nucleosome sliding by preventing both thermally driven passive mobility and SNF2h-mediated remodeling. These results indicate that even a single DHC is sufficient to rigidify the nucleosome, suppressing molecular fluctuations and reinforcing DNA–histone interactions throughout the entire particle. Importantly, DHC formation does not disrupt the overall architecture of the NCP, providing a unique means to stabilize nucleosome substrates without compromising their structural integrity. This property makes cross-linked nucleosomes valuable tools for biochemical and biophysical applications, such as generating stable NCP–nuclear factor complexes for high-resolution cryo-electron microscopy.

We further show that DHC formation increases the transcriptional barrier for SP6 RNA polymerase. The presence of a DHC not only impedes transcription elongation but also prevents transcription-induced nucleosome translocation, leading to premature transcription termination approximately 15 bp upstream of the cross-link site. Moreover, DHCs render histones more resistant to proteolysis. Together, these findings establish DHCs as a highly toxic and persistent form of DNA damage. This combination of persistence and functional disruption may render DHC lesions particularly deleterious in cancer, suggesting that reagents capable of selectively inducing DHCs could represent a promising avenue for anticancer therapy.

To our knowledge, this work represents the first systematic investigation of how DHCs affect nucleosome structure and function *in vitro*. Our findings suggest that DHC formation broadly interferes with the recognition and processing of nucleosomes by diverse nuclear factors. It is therefore plausible that DHCs may also hinder other essential chromatin-based processes, including DNA replication, repair, and epigenetic regulation. Elucidating the cellular consequences of DHCs in eukaryotic systems remains an important direction for future research.

## Supplementary Material

pwaf094_Supplementary_Data

## Footnotes

The authors thank Professor Zhucheng Chen (Tsinghua University) for providing the SNF2h plasmid.

This work was supported by the National Natural Science Foundation of China (No. 22377059 to C.Z., and No. 22407065 to Y. Z.), the Natural Science Foundation of Tianjin Municipality (No. 24JCYBJC01930 to C.Z.), National Key Research and Development Program of China (No. 2023YFA0913800 to C.Z.), and Haihe Laboratory of Sustainable Chemical Transformations.

The authors declare no competing interests.

The structural model of the nucleosome core particle with site-specific DNA–histone cross-link (DHC) generated in this study has been deposited in the Protein Data Bank (PDB) under accession code 9UZ7. The corresponding cryo-EM reconstructions of nucleosome core particle with site-specific DHC have been deposited in the Electron Microscopy Data Bank (EMDB) under accession code EMD-64640.

X.S. performed most of the biochemical experiments, as well as sample preparation for cryo-EM and data collection. G.J. and H.L. carried out the cryo-EM data processing and structure refinement. J.L., M.R., and J.M. participated in the biochemical experiments. Y.Z. contributed new reagents and analytical tools. C.Z. and H.L. designed and supervised the project. X.S., G.J., H.Li., and C.Z. wrote the manuscript. All authors have given approval to the final version of the manuscript.
