# Supplementary material for: DNA–histone cross-link locks the nucleosome structure and disrupts its recognition and processing
Source: Protein Cell. 2025 Nov 5;17(5):476–82. doi: 10.1093/procel/pwaf094 (PMC13161471; doi:10.1093/procel/pwaf094)
Supplement: pwaf094_Supplementary_Data [file pwaf094_supplementary_data.zip › Supplementary Materials.pdf]

Materials and Methods

Materials

Native oligonucleotides were purchased from Shanghai Sangon Biotech Co. Ltd. ssDNAs were prepared by ligating short oligonucleotides (Fig. S1-3), as previously described (Li et al., 2017). The synthesis of octadiynyl-dU phosphoramidite was performed according to established protocols (Srinivasan et al., 2001). Nucleosome core particles (NCPs) without fluorophore modification were used in the thermal stability assay. For the *in vitro* transcription assay, the octadiynyl-dU modified strand was labeled with 5'-Cy5, while 5'-FAM labeling was used in all other experiments. Histone expression and purification were performed as previously described (Kujirai et al., 2018; Zhou et al., 2013). The plasmid for SNF2h expression was generously provided by Professor Zhucheng Chen (Tsinghua University), and SNF2h was expressed and purified according to reported procedures (Yan et al., 2019). IA-PEG$_4$-N$_3$ and BTTAA were purchased from Confluore Biological (Xi'an, China). SP6 RNA polymerase and *E. coli* Poly (A) polymerase were purchased from Beyotime (Shanghai, China). BamHI and BanI were purchased from NEB. Gels were visualized with an Amersham Typhoon Gel and Blot Imaging System.

IA-PEG$_4$-N$_3$ labeling of histone H3.

A total of 130 nmol of histone H3 (C96/S, C110/S, K115/C) was dissolved in 280 μL of HEPES buffer-1 (7 M guanidine hydrochloride, 20 mM HEPES, pH 8.0). The solution was incubated with 5 equivalents of TCEP at room temperature for 30 minutes to reduce disulfide bonds. Excess TCEP was removed using a PD SpinTrap G-25 column, following the manufacturer's instructions. Subsequently, 10 equivalents of IA-PEG$_4$-N$_3$ were added, and the reaction mixture was incubated at room temperature for 45 minutes in the dark. Labeling efficiency was assessed by MALDI-TOF mass spectrometry. For desalting, the sample was dialyzed against 2 L of water containing 2 mM 2-mercaptoethanol, with the dialysis performed three times for 12 hours each. The desalted sample was then lyophilized for subsequent octamer reconstitution.

Reconstitution of nucleosomes.

The general procedure for nucleosome reconstitution has been described previously (Li et al., 2017). Briefly, double-stranded DNA (100 pmol) and histone octamer (100 pmol) were mixed in a Slide-A-Lyzer MINI Dialysis Unit (3,500 MWCO, Thermo Scientific, Cat. No. 69550) containing 100 μL of an aqueous solution with 2 M NaCl. The dialysis unit was placed inside a dialysis bag filled with 20 mL of high-salt buffer (2 M NaCl, 10 mM HEPES, pH 7.5, 0.1 mM PMSF), and the bag was subsequently immersed in 2 L of low-salt buffer (10 mM HEPES, pH 7.5, 0.1 mM PMSF) for overnight dialysis at 4 °C. Any precipitate formed during dialysis was removed by centrifugation at 2,000× g for 10 minutes. The resulting supernatant was transferred to a fresh siliconized tube. To assess reconstitution efficiency, a small aliquot was analyzed by native nucleoprotein gel electrophoresis (10 × 8 × 0.15 cm; 5% (w/v) acrylamide/bisacrylamide, 59:1; 0.6× TBE running buffer; run at 4 °C using 0.2× TBE buffer). For thermal stability experiments, NCPs were further purified using a Model 491 Prep Cell as previously reported (Dyer et al., 2003). All recombinant NCPs were stored at 4 °C and used directly in subsequent experiments. Nucleosome concentrations were determined based on the A_{260} of the associated DNA.

CuAAC reaction.

Copper-catalyzed azide–alkyne cycloaddition (CuAAC) within nucleosomes was performed following previously reported protocols (Besanceney-Webler et al., 2011). To the NCP solution (10 pmol in 10 mM HEPES buffer, pH 7.5, 20 mM NaCl, 0.1 mM PMSF), $CuSO_4$/BTTAA (final concentration: 50 μM; molar ratio $CuSO_4$:BTTAA = 1:6) and ascorbate (final concentration: 2.5 mM) were added. The total reaction volume was adjusted to 200 μL and incubated at 37 °C for 2 hours. To monitor DHC formation, a 10 μL aliquot was taken and analyzed by 10% SDS-PAGE. To assess the structural integrity of NCPs, a separate 5 μL aliquot was analyzed by 5% native PAGE.

Cryo-EM sample preparation and data collection.

The sample was dialyzed three times for 6 hours each to sample buffer (20 mM HEPES, 1 mM DTT) and concentrated to 80 ng/μL using MWCO 30 kDa. A 3.5 μl aliquot of the nucleosome particle was deposited

onto glow-discharged Quantifoil gold grids (R1.2/1.3 300-mesh Au) for 30 seconds. The grids were then blotted for 3.5 seconds with force of -2 using a Vitrobot Mark IV (FEI Company) under conditions of 8 °C and 100% humidity, followed by rapid plunge-freezing in liquid ethane.

A total of 1,978 micrographs were acquired using the EPU auto-acquisition software on a Thermo Fisher Scientific Titan Krios G3i (300 kV) TEM, equipped with a Gatan K3 direct electron detector and a GIF-Quantum energy filter. Data were collected with defocus values ranging from −1.0 to −2.0 μm and a pixel size of 0.54 Å. Each micrograph was fractionated into 32 frames, with a total irradiation dose of approximately 50 e-/Å^2.

Cryo-EM data processing, model building and refinement.

The raw movies were imported into CryoSPARC (Punjani et al., 2017) for patch motion correction and CTF estimation. Nucleosome particles were automatically picked using the blob picker tool in CryoSPARC and extracted into 400 × 400 pixel boxes with 2 × binning. After 2D classification, a total of 1,081,772 particles were selected. An ab initio 3D model was generated with C1 symmetry and subsequently refined through 3D classification. One class, comprising 199,160 particles, was selected for non-uniform refinement, CTF refinement, and particle polishing in CryoSPARC. The final reconstruction, representing a well-defined nucleosome structure, was resolved to a global resolution of 3.24 Å after solvent mask post-processing. Resolutions were reported at the 0.143 Fourier shell correlation cut-off using gold-standard refinement (Rosenthal and Henderson, 2003).

For atomic model building, the PDB structure 8JBX was rigid-body fitted into the cryo-EM density map of the cross-linked nucleosome using UCSF ChimeraX (Pettersen et al., 2004). Local adjustments of secondary structure elements and side chains to the densities were performed in Coot (Emsley and Cowtan, 2004). The cross-linked C115 molecule was generated using the Coot Ligand Builder and substituted for C115 in the model. DNA and histone sequences from PDB 8JBX were mutated to match the sequences used in this study (histone and 601 DNA). Model refinement was conducted in PHENIX (phenix.real_space_refine)

(Adams et al., 2010). The refined model was visually inspected, and Ramachandran outliers and other problematic regions were manually corrected in Coot. Model validation statistics are provided in the Table S2.

Stability of NCP in high-salt conditions.

^{N3}NCP^{Alkynyl} and cross-linked NCP (240 ng/μL) were incubated in the presence of 0, 0.4, 0.6, 0.8, and 1 M NaCl at 55 °C for 1 h. Following incubation, the NaCl concentration in all samples were adjusted to 0.4 M. Samples were then subjected to 5% native PAGE to assess NCP stability.

NCP dilution assay.

A 2 μM solution of ^{N3}NCP^{Alkynyl} or cross-linked NCP was prepared and divided into 25 μL aliquots. Each aliquot was diluted using HEPES 20 buffer [10 mM HEPES (pH 7.5), 20 mM NaCl] to generate a dilution series. The samples were incubated at 4 °C for 24 hours and subsequently reconcentrated to 25 μL using 30 kDa molecular weight cut-off (MWCO) concentrators (Amicon, Sartorius). The samples were analyzed by 5% native PAGE to assess NCP integrity.

Thermal stability assay.

The thermal stabilities of ^{N3}NCP^{Alkynyl} and cross-linked NCPs were evaluated using a thermal shift assay, as previously described (Taguchi et al., 2014). NCP samples (2.25 μM) were incubated in a reaction mixture containing HEPES 20 buffer [10 mM HEPES (pH 7.5), 20 mM NaCl] and SYPRO Orange Protein Gel Stain (Sigma-Aldrich) diluted 1:1000. The temperature was gradually increased from 25 °C to 95 °C at a rate of 1 °C per minute, and fluorescence was monitored using a real-time PCR system (Bio-Rad). Fluorescence intensity was plotted as a function of temperature to assess thermal stability.

Thermal sliding of nucleosomes.

Samples of 40-^{N3}NCP^{Alkynyl}-40 or cross-linked 40-NCP-40 (1 pmol in 10 μL) were incubated at 60 °C to induce thermal sliding. Aliquots were collected at designated time intervals, and sucrose was added to each to a final concentration of 5% (w/v). The reaction mixtures were then immediately placed on ice to terminate the reaction. Samples were analyzed by 7% native PAGE, run in 0.2 × TBE buffer at 180 V for 90 minutes at 4 °C.

SNF2h-driven nucleosome sliding.

Nucleosome sliding assays were performed as previously described(Yan et al., 2019), using 40 nM ^{N3}NCP^{Alkynyl}-80 or cross-linked NCP-80 and varying concentrations of SNF2h (0, 0.05, 0.1, 0.2, 0.4, and 0.8 μM). Reactions were carried out in buffer containing 50 mM KCl, 20 mM HEPES (pH 7.5), 5 mM $MgCl_2$, 0.1 mg/mL bovine serum albumin, 5% glycerol, and 1 mM DTT, supplemented with 2 mM ATP. Samples were incubated at 37 °C for 32 minutes and quenched by the addition of 150 ng sperm DNA. Reaction products were resolved on 7% native polyacrylamide gels in 0.25 × TBE buffer at 4 °C, run at 150 V for 120–180 minutes.

EMSA analysis of SNF2h binding to nucleosomes.

Ten nanomolar ^{N3}NCP^{Alkynyl} and cross-linked NCPs were mixed with increasing concentrations of SNF2h in 15-μL reactions containing 50 mM KCl, 20 mM HEPES (pH 7.5), 5 mM $MgCl_2$, 0.1 mg/mL bovine serum albumin, 5% glycerol, and 1 mM DTT. After incubation on ice for 30 min, samples were resolved by electrophoresis on 5% native acrylamide gels in 0.2× TBE buffer at 4 °C for 45 minutes. Bound fractions were quantified based on the disappearance of free NCPs relative to the total band intensity in each lane.

Transcription of nucleosomes.

Before transcription, the binding of SP6 RNA polymerase to nucleosomes was verified by EMSA. Specifically, 30 ng of uncrosslinked SP6-^{N3}NCP$^{Alkynyl\text{-}AS}$ and cross-linked SP6-nucleosome^{AS} were incubated with 40 U of SP6 RNA polymerase in a 20 μL reaction containing 40 mM Tris-HCl (pH 7.9), 2 mM spermidine, 6 mM $MgCl_2$, 1 mM DTT, 40 mM KCl, and 0.1 mg/mL BSA. After incubation on ice for 30 min, the samples were resolved by electrophoresis on 5% native acrylamide gels in 0.2× TBE buffer for 45 minutes at 4 °C.

Transcription assays were performed following previously reported protocols (Studitsky et al., 1994, 1995). Briefly, 30 ng of template DNA in a 20 μL reaction volume was transcribed using 40 U of SP6 RNA Polymerase (20 U/μL, Beyotime) in a buffer containing 40 mM Tris-HCl (pH 7.9), 2 mM spermidine, 6 mM $MgCl_2$, 1 mM DTT, 40 mM KCl, 0.1 mg/mL BSA, 1 U of RNase inhibitor, and 0.5 mM NTPs. Reactions were

incubated at 37 °C for 30 minutes. To monitor the structural integrity of the nucleosome core particles (NCPs), a 5 μL aliquot was removed and analyzed by 5% native PAGE.

For experiments involving restriction digestion of the templates before and after transcription, 10 U of the appropriate restriction enzyme was added, followed by incubation at 37 °C for 30 minutes. The reaction was quenched by adding NaEDTA (pH 7.0) to a final concentration of 10 mM. Each 15 μL aliquot was then treated with 1 μL of proteinase K (0.4 U), followed by the addition of formamide (1:1 volume ratio). The samples were heated at 95 °C for 5 minutes and subsequently analyzed by 8%/20% two-layer denaturing PAGE.

3'RACE analysis of transcripts.

10 U of DNAse I (NEB) was added to the transcription reaction and incubated at room temperature for 30 min to degrade the DNA template. Poly(A) tailing was then performed by adding ATP to a final concentration of 1 mM and E. coli Poly(A) Polymerase (100 nM), followed by incubation at 37 °C for 30 minutes. The reaction was quenched by heating at 65 °C for 5 minutes and then cooled on ice for 5 minutes.

First-strand cDNA synthesis was carried out using the Hifair® III 1st Strand cDNA Synthesis SuperMix (Yeasen Biotech) according to the manufacturer’s instructions. Subsequent PCR amplification was performed using the TransStart GoldPfu PCR SuperMix. The primers used for 3' RACE RT-PCR were as follows:

Forward primer: 5'- GTTGTAAAACGACGGCCAG GCGCGTACGTGCGTTTAAGC-3',

Reverse primer: 5'- GCTGTCAACGATACGCTACGCTAACGGCATGACAGTGTTTTTTTTTTTTTTTTT TTT-3'.

Following RT-PCR amplification, the double-stranded DNA products were separated by 3% agarose gel electrophoresis. Target bands were excised and purified using a gel extraction kit (Tianmobio, Beijing, China). The purified PCR products were then subjected to cloning using the pEASY®-Blunt3 Cloning Kit (TransGen Biotech), following the manufacturer’s protocol. Finally, one-quarter of the cloned colonies were selected and submitted to Tsingke Biotech (Tianjin, China) for Sanger sequencing.

Preparation of free DHC.

A 1 mL reaction mixture containing 20 mM HEPES buffer (pH 7.5), 145 nt ssDNA (f-601 DNA-X^{74}, 0.5 nmol), H3-azide (2.5 nmol), $CuSO_4 \cdot 5H_2O$ (0.5 mM), BTTAA (3 mM), and sodium ascorbate (25 mM) was incubated at 37 °C for 2 h. After incubation, urea was added to a final concentration of 2 M, followed by mixing with an equal volume of loading buffer (50 mM HEPES, pH 7.5, 40% glycerol, 0.2% SDS). The reaction products were purified by 10% SDS-PAGE. The desired band was excised, crushed, and incubated with elution buffer (25 mM Tris, pH 8.3, 192 mM glycine, 0.1% SDS) for electroelution. The eluate was centrifuged, and the supernatant was carefully collected and desalted by repeated buffer exchange into 20 mM HEPES (pH 7.5) using a 3-kDa cut-off Amicon filter. To generate free DHC, an equimolar amount of the complementary reverse oligonucleotide (145 nt) was added to the ssDHC, and annealing was carried out by heating the mixture to 95 °C for 2 min followed by gradual cooling to room temperature.

Trypsin degradation assay.

Cross-linked NCP and free DHC (2.5 pmol each) were incubated with 50 ng of trypsin at 37 °C in 100 µL of reaction buffer containing 50 mM Tris-HCl (pH 7.9) and 20 mM $CaCl_2$. At designated time points (1, 5, 15, 30, and 60 minutes), 20 µL aliquots were withdrawn, and the reaction was quenched by adding SDS loading buffer. The digestion products were resolved by 10% SDS-PAGE.

Proteinase K degradation assay.

${}^{N3}NCP^{Alkynyl}$ and cross-linked NCP (2 pmol) was incubated with 0.4 ug of proteinase K at 37 °C in 20 µL of reaction buffer containing 20 mM HEPES (pH 7.5) and 20 mM NaCl. Aliquots (5 µL) were collected at 0, 5, 30, and 60 minutes, and the digestion products were analyzed by 5% native PAGE.

Table S1. DNA sequences used for nucleosome preparation.

**Entry**	**Sequence**	
f601-F1	(FAM)-ATCGATGTATATATCTGACACGTGCCTGGA (30 nt)	X = 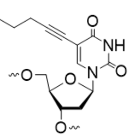
601-F1	ATCGATGTATATATCTGACACGTGCCTGGA (30 nt)	
p601-F1	pATCGATGTATATATCTGACACGTGCCTGGA (30 nt)	
p601-F2	pGACTAGGGAGTAATCCCCTTGGCGGTTAAAACGCG (35 nt)	
p601-F3	pGGGGACAGCGCGTACGTGCGTTTGAGCGGTGCTAG (35 nt)	
p601-F4	pAGCTGTCTACGACCAATTGAGCGGCCTCGGCACCGGGATTCTGAT (45 nt)	
601-T1	CTCCCTAGTCTCCAGGCACG (20 nt)	p = phosphate
601-T2	CGCTGTCCCCCGCGTTTTAA (20 nt)	
601-T3	GTAGACAGCTCTAGCACCGC (20 nt)	
f601-R1	(FAM)-ATCAGAATCCCGGTGCCGAGGCCGCTCAATTGGTC (35 nt)	
601-R1	ATCAGAATCCCGGTGCCGAGGCCGCTCAATTGGTC (35 nt)	
p601-R1	pATCAGAATCCCGGTGCCGAGGCCGCTCAATTGGTC (35 nt)	
p601-R2	pGTAGACAGCTCTAGCACCGCTCAAACGCAC (30 nt)	
p601-R3U^{74}	pGTACGCGC**X**^{74}GTCCCCCGCGTTTTAACCGCCAAGGG (35 nt)	
p601-r3U^{74}	pGTACGCGC**X**^{74}GTCCCCCG (17 nt)	
p601-r3B	pCGTTTTAACCGCCAAGGG (18 nt)	
p601-R4	pGATTACTCCCTAGTCTCCAGGCACGTGTCAGATATATACATCGAT (45 nt)	
601-T4	AGCTGTCTACGACCAATTGA (20 nt)	
601-T5	CAGCGCGTACGTGCGTTTGA (20 nt)	
601-T6	GGGAGTAATCCCCTTGGCGG (20 nt)	
601-t1	GGTTAAAACGCGGGGGACAG (20 nt)	
40-601-40-A	(FAM)-AGTTCATCCCTTATGTGATGGACCCTATACGCGGCCGCCC (40 nt)	
40-601-40-B	pTGCATGTATTGAACAGCGACCTTGCCGGTGCCAGTCGGAT (40 nt)	
40-601-40-T1	ACCGGGATTCTGATGGGCGGCCGCGTAT (28 nt)	
40-601-40-T2	GTTCAATACATGCAATCGATGTATATAT (28 nt)	
40-601-40-C	ATCCGACTGGCACCGGCAAGGTCGCTGTTCAATACATGCA (40 nt)	
40-601-40-D	pGGGCGGCCGCGTATAGGGTCCATCACATAAGGGATGAACT (40 nt)	
40-601-40-T3	ATATATACATCGATTGCATGTATTGAAC (28 nt)	
40-601-40-T4	ATACGCGGCCGCCCATCAGAATCCCGGT (28 nt)	
80-601-A	(FAM)-GGGATCCTAATGACCAAGGAAAGCATGATTCTTCACACCGAGTTCATC CCTTATGTGATGGACCCTATACGCGGCCGCCC (80 nt)	
80-601-B	pGGGCGGCCGCGTATAGGGTCCATCACATAAGGGATGAACTCGGTGTGAAGAA TCATGCTTTCCTTGGTCATTAGGATCCC (80 nt)	
80-601-T1	GGATTCTGATGGGCGGCCGC (20 nt)	
80-601-T2	GCGGCCGCCCATCAGAATCC (20 nt)	
p601-f3U^{125}	pGGGGAC**X**^{125}GCGCGTACGT (17 nt)	
p601-f3B	pGCGTTTGAGCGGTGCTAG (18 nt)	
p601-F3U^{125}	pGGGGAC**X**^{125}GCGCGTACGTGCGTTTGAGCGGTGCTAG (35 nt)	
Cy5-601-R1	(Cy5)-ATCAGAATCCCGGTGCCGAGGCCGCTCAATTGGTC (35 nt)	
p601-R3^{125}	pGTACGCGCAGTCCCCCGCGTTTTAACCGCCAAGGG (35 nt)	
601-t2	GCTTAAACGCACGTACGCGC (20 nt)	

SP6-601-A	pGGTGTCGCTTTCGTGGATCCCCATTAACTCTTCTATAGTGTCACCTAAATCGT (53 nt)	
SP6-601-B	ACGATTTAGGTGACACTATAGAAGAGTTAATGGGGATCCACGAAAGCGACACC (53 nt)	
Cy5-*SP6*-601-B	(Cy5) ACGATTTAGGTGACACTATAGAAGAGTTAATGGGGATCCACGAAAGCGACACC (53 nt)	
SP6-601-T1	CACGAAAGCGACACCATCGATGTATATATC (20 nt)	
SP6-601-T2	GATATATACATCGATGGTGTCGCTTTCGTG (20 nt)	
145 nt 601 DNA	5'(FAM)ATCAGAATCCCGGTGCCGAGGCCGCTCAATTGGTCGTAGACAGCTCTAGCACCGCTCAAACGCACGTACGCGC**X**^{74}GTCCCCCGCGTTTTAACCGCCAAGGGGATTACTCCCTAGTCTCCAGGCACGTGTCAGATATATACATCGAT	
cDNA of 145 nt 601 DNA	5'ATCGATGTATATATCTGACACGTGCCTGGAGACTAGGGAGTAATCCCCTTGGCGGTTAAAACGCGGGGGACAGCGCGTACGTGCGTTTGAGCGGTGCTAGAGCTGTCTACGACCAATTGAGCGGCCTCGGCACCGGGATTCTGAT3'	

Table S2. Cryo-EM data collection, refinement and validation statistics.

Sample	Cross-linked NCP (EMDB: 64640) (PDB: 9UZ7)
**Data collection and processing**	
Magnification	64 000x
Voltage (kV)	300
Electron exposure (e-/Å²)	50
Defocus range (μm)	-1.0 to -2.0
Pixel size (Å)	1.08
Symmetry imposed	C1
Initial particle images (no.)	1,081,772
Final particle images (no.)	199,160
Map resolution (Å)	3.27
FSC threshold	0.143
**Refinement**	
Initial model used (PDB code)	8JBX
Model resolution (Å)	3.4
FSC threshold	0.143
Model composition	
Non-hydrogen atoms	11817
Protein residues	765
Nucleotides	280
Ligands	1
B factors (Å)	
Protein	0.00/57.98/9.71
Nucleotides	21.48/108.66/62.01
Ligand	1.68/1.68/1.68
R.m.s. deviations	
Bond lengths (A)	0.004
Bond angles (°)	0.714
Validation	
MolProbity score	1.04
Clashscore	1.73
Poor rotamers (%)	0.47
Ramachandran plot	
Favored (%)	97.46
Allowed (%)	2.54
Disallowed (%)	0.00

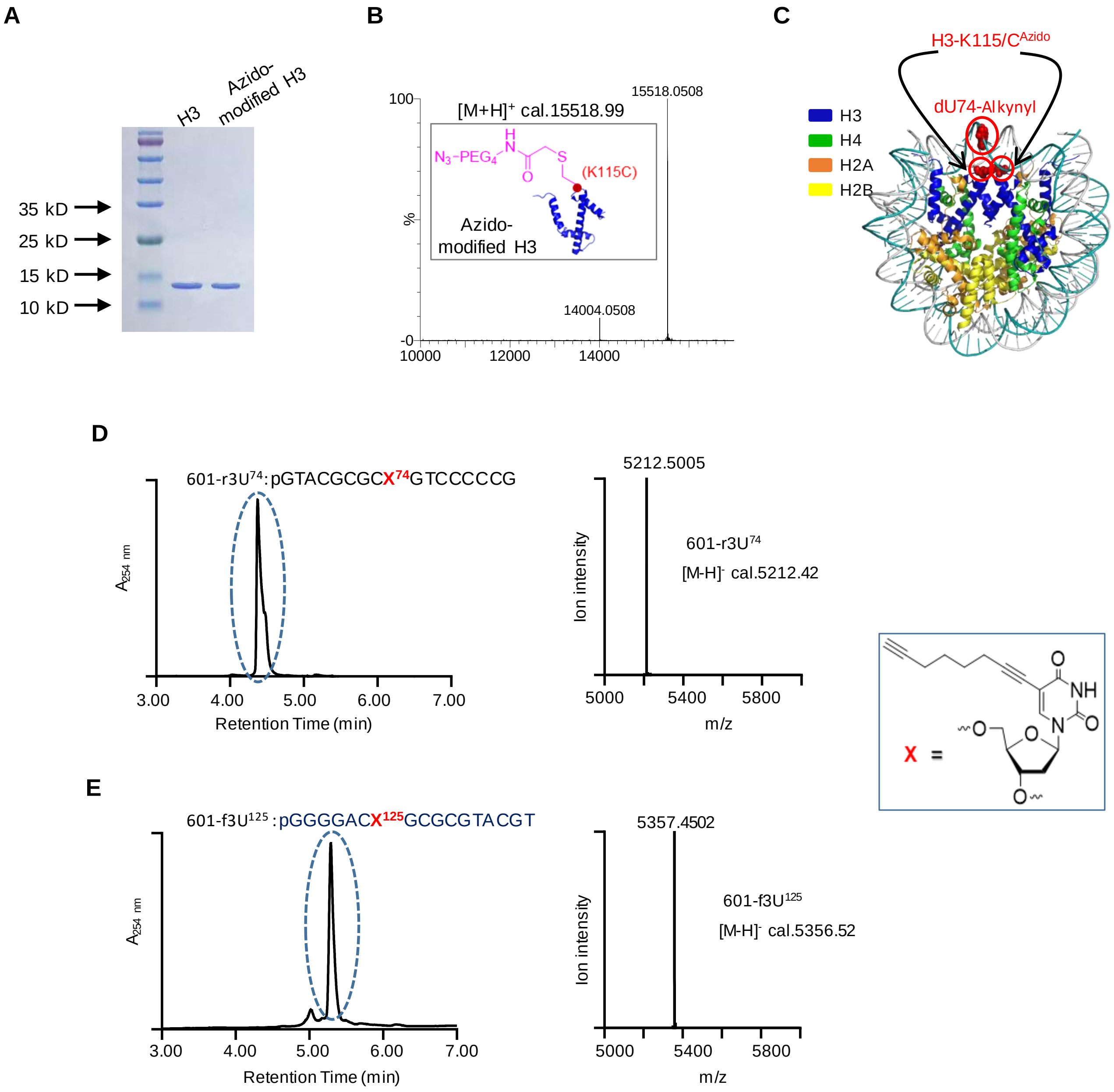

Figure S1. **Characterization of azido-modified H3 and alkynyl-modified DNA.** (A) 15% SDS-PAGE analysis showing the purity of azido-modified H3. (B) Mass spectrometry analysis of azido-modified H3. (C) X-ray crystal structure of the nucleosome core particle (PDB: 3LZ0) showing the positions of dU74-alkynyl and H3-K115C-azido modifications. (D) UPLC-MS analysis of the alkynyl-modified DNA 601-r3U^{74}. The mass spectrum corresponding to the circled chromatographic peak is shown in the right panel. (E) UPLC-MS analysis of the alkynyl-modified DNA 601-f3U^{125}. The mass spectrum corresponding to the circled chromatographic peak is shown in the right panel.

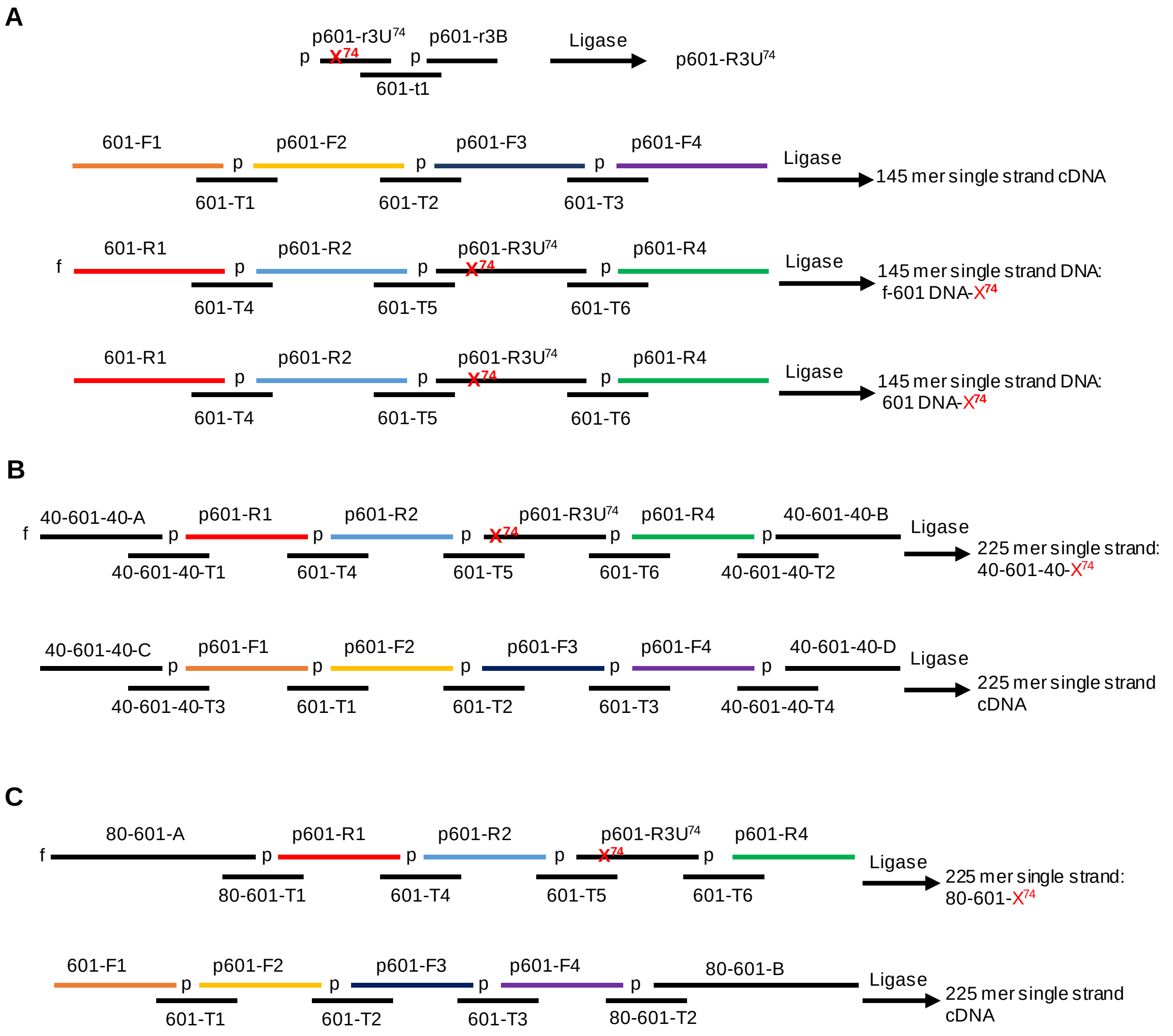

Figure S2. Preparation of dsDNA for nucleosome assembly in structural studies. (A) Ligation scheme for generating 145 bp dsDNA used in nucleosome core particle (NCP) assembly. (B) Ligation scheme for generating 225 bp dsDNA used in assembling nucleosome 40-^{N3}NCP^{Alkynyl}-40. (C) Ligation scheme for generating 225 bp dsDNA used in assembling nucleosome ^{N3}NCP^{Alkynyl}-80.

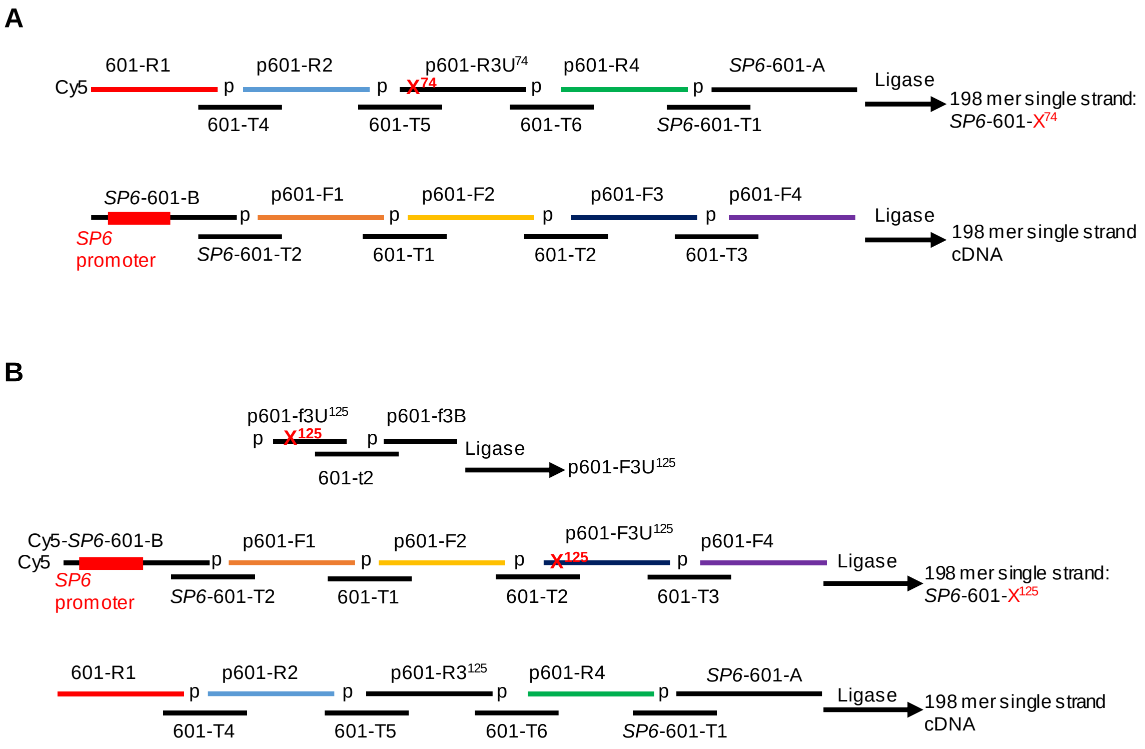

Figure S3. Preparation of dsDNA for assembly of nucleosomes SP6-^{N_3}NCP$^{Alkynyl\text{-}AS}$ and SP6-^{N_3}NCP$^{Alkynyl\text{-}SS}$. (A) Ligation scheme for generating 198 bp dsDNA used in assembling nucleosome SP6-^{N_3}NCP$^{Alkynyl\text{-}AS}$. (C) Ligation scheme for generating 198 bp dsDNA used in assembling nucleosome SP6-^{N_3}NCP$^{Alkynyl\text{-}SS}$.

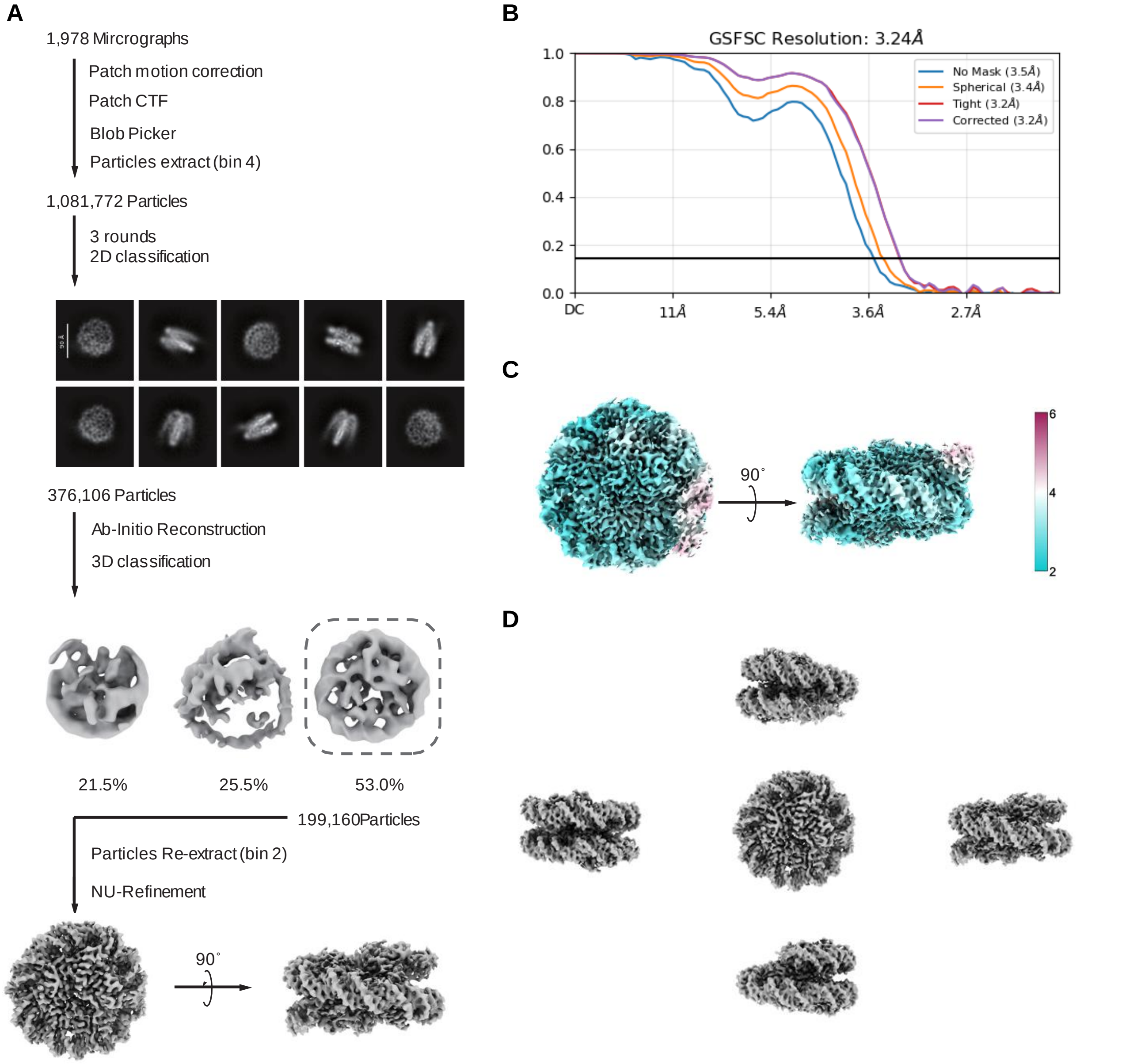

Figure S4. The cross-linked nucleosome Cryo-EM data processing. (A) Flow chart of the cryo-EM data processing procedure. (B) Fourier Shell Correlation (FSC) curve of the cross-linked NCP density map. The final resolution is 3.24 Å. (C) Local resolution map of the cross-linked NCP. (D) Cryo-EM density map of the cross-linked NCP. Disc view (middle) and gyre views (left, right, top and bottom).

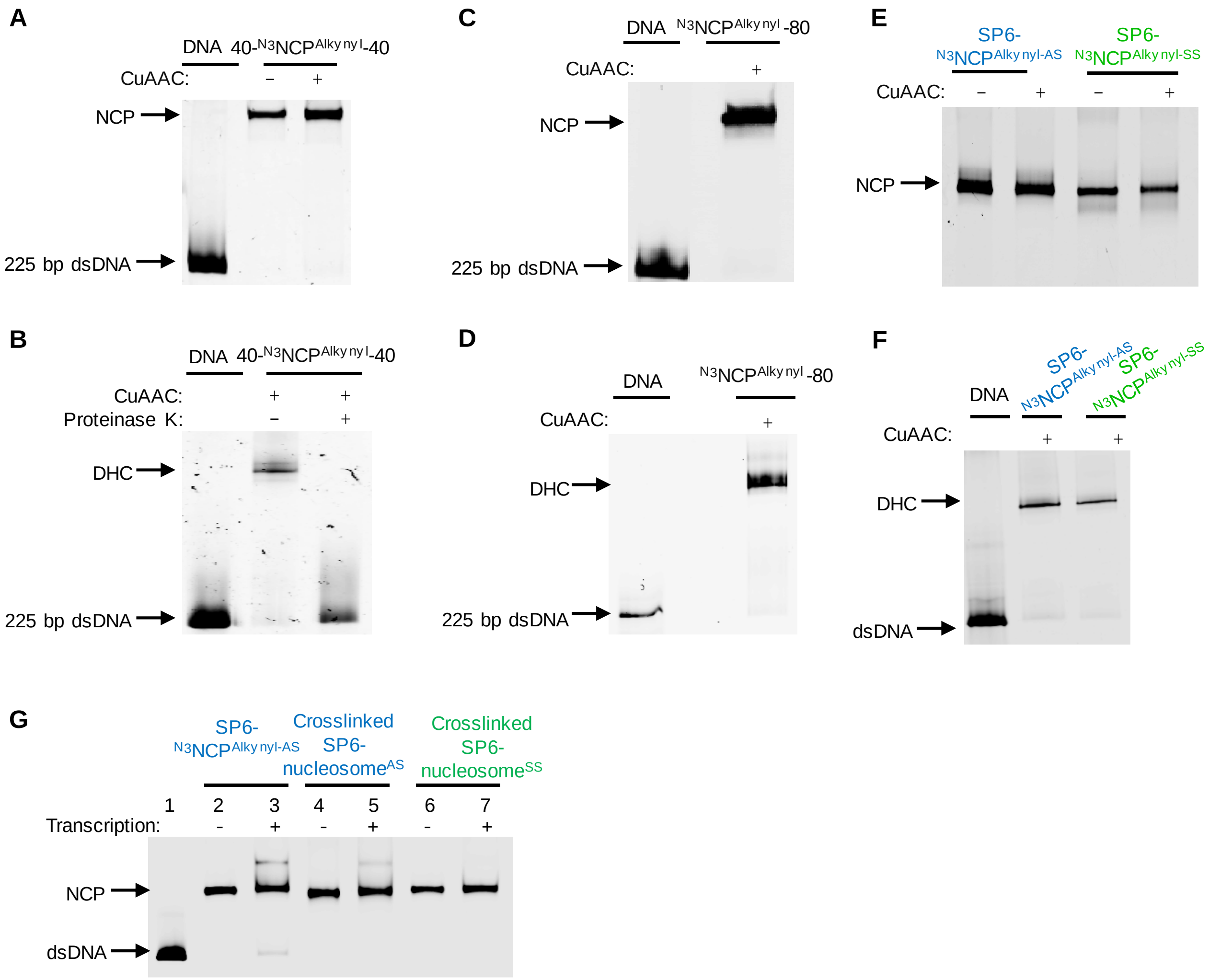

Figure S5. PAGE analysis demonstrating the structural integrity of cross-linked and uncrosslinked nucleosomes. (A) 5% native PAGE analysis demonstrating the structural integrity of uncrosslinked nucleosome 40-^{N3}NCP^{Alkynyl}-40 and cross-linked 40-NCP-40. (B) 10% SDS-PAGE analysis showing the formation of DHCs in nucleosome 40-^{N3}NCP^{Alkynyl}-40 via CuAAC. (C) 5% native PAGE analysis demonstrating the structural integrity of nucleosome ^{N3}NCP^{Alkynyl}-80. (D) 10% SDS-PAGE analysis showing the formation of DHCs in nucleosome ^{N3}NCP^{Alkynyl}-80 via CuAAC. (E) 5% native PAGE analysis demonstrating the structural integrity of SP6-^{N3}NCP$^{Alkynyl-AS}$ and SP6-^{N3}NCP$^{Alkynyl-SS}$. (F) 10% SDS-PAGE analysis showing the formation of DHCs in nucleosomes SP6-^{N3}NCP$^{Alkynyl-AS}$ and SP6-^{N3}NCP$^{Alkynyl-SS}$ via CuAAC. (G) Native PAGE (5%) analysis of nucleosomes before and after transcription.

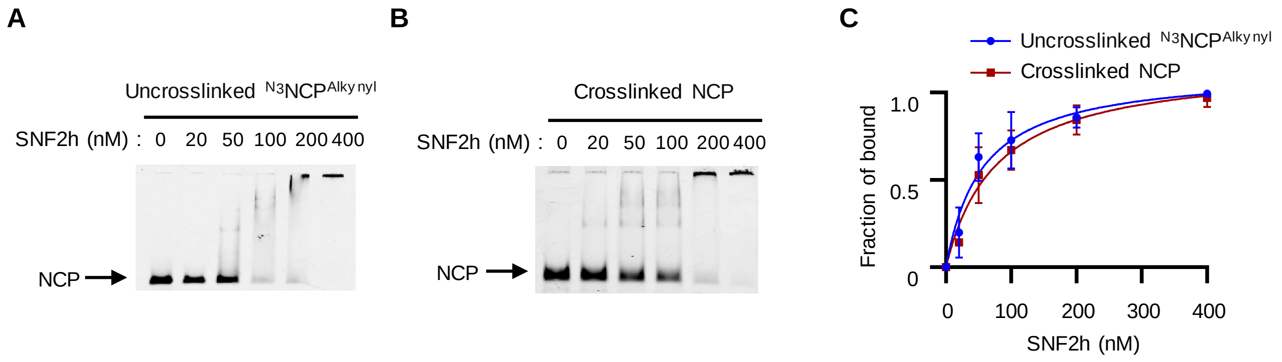

Figure S6. **EMSA analysis demonstrating the binding of SNF2h to cross-linked and uncrosslinked nucleosomes.** (A) 5% native PAGE showing the binding of SNF2h to the uncrosslinked nucleosome $^{N3}NCP^{Alkynyl}$. (B) 5% native PAGE showing the binding of SNF2h to the cross-linked nucleosome. (C) Plot of the fraction of bound nucleosomes versus the concentration of SNF2h.

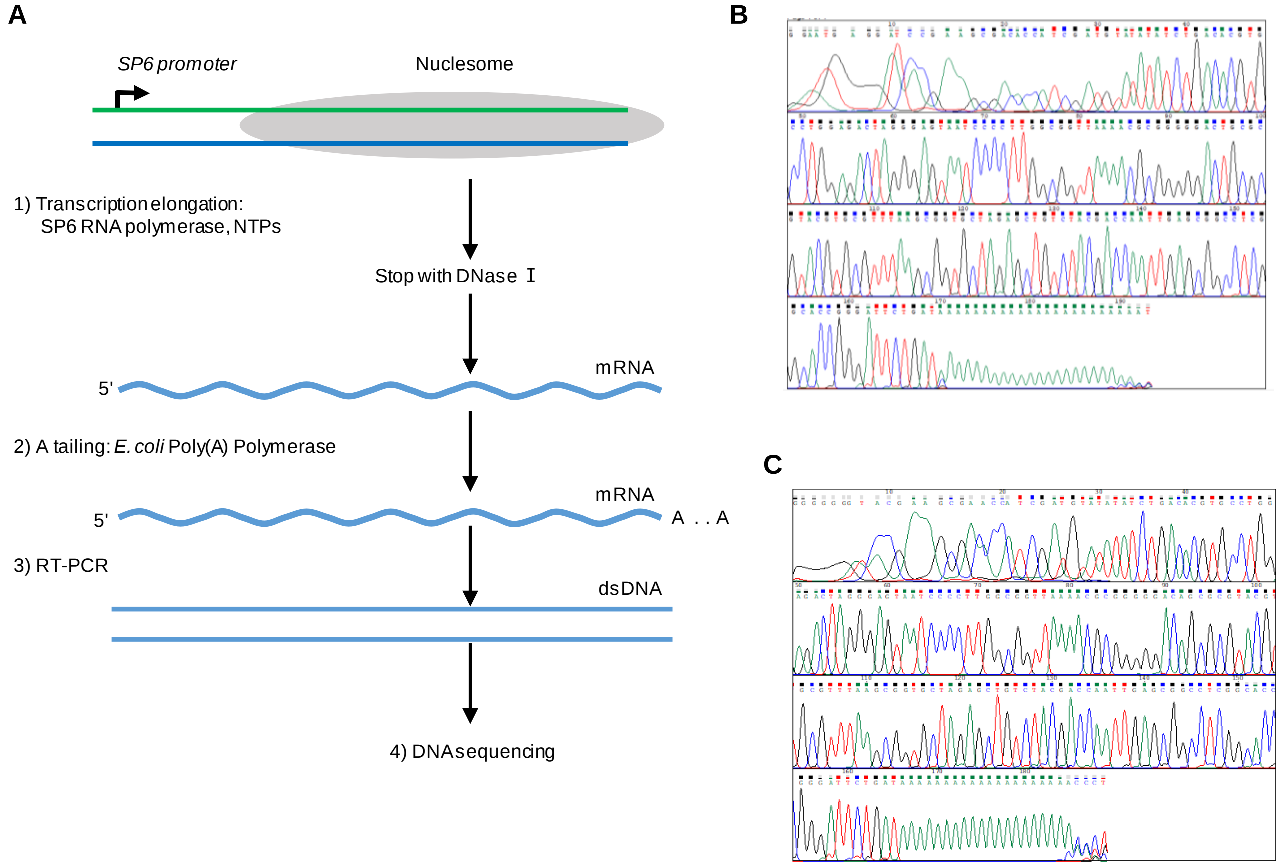

Figure S7. Analysis of transcripts generated by SP6 RNA polymerase-mediated transcription within nucleosomes. (A) The strategy of 3' RACE analysis of transcripts. (B) Sequencing profile of the transcript from SP6-^{N_3}NCP$^{Alkynyl\text{-}SS}$. (C) Sequencing profile of the transcript from SP6-^{N_3}NCP$^{Alkynyl\text{-}AS}$.

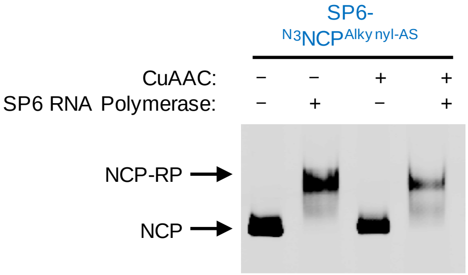

Figure S8. EMSA analysis of the binding of SP6 RNA polymerase to cross-linked and uncrosslinked nucleosomes.

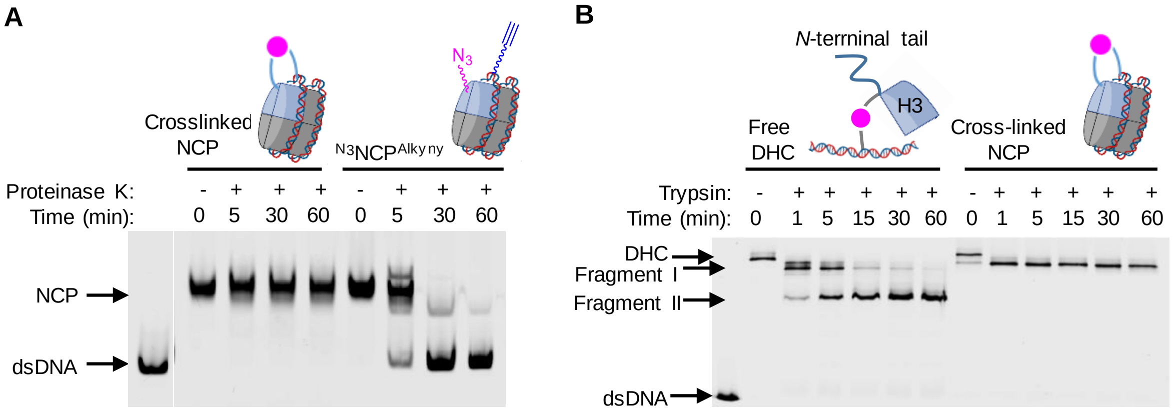

Figure S9. Protease-mediated degradation of histones in different substrates. (A) Native 5% PAGE analysis of nucleosomes following treatment with proteinase K. (B) 10% SDS-PAGE analysis of trypsin-mediated degradation of free DNA-H3 cross-links and cross-linked NCP.

Supplementary References

Adams, P.D., Afonine, P.V., Bunkoczi, G., *et al.* PHENIX: a comprehensive Python-based system for macromolecular structure solution. Acta Crystallogr, Sect D: Biol Crystallogr 2010;**66**:213-221.

Besanceney-Webler, C., Jiang, H., Zheng, T., *et al.* Increasing the efficacy of bioorthogonal click reactions for bioconjugation: A comparative study. Angew Chem Int Ed 2011;**50**:8051-8056.

Dyer, P.N., Edayathumangalam, R.S., White, C.L., *et al.* (2003). Reconstitution of nucleosome core particles from recombinant histones and DNA. In Methods Enzymol (Academic Press), pp. 23-44.

Emsley, P., and Cowtan, K. Coot: model-building tools for molecular graphics. Acta Crystallogr, Sect D: Biol Crystallogr 2004;**60**:2126-2132.

Kujirai, T., Arimura, Y., Fujita, R., *et al.* Methods for preparing nucleosomes containing histone variants. Methods Mol Biol 2018;**1832**:3-20.

Li, F., Zhang, Y., Bai, J., *et al.* 5-Formylcytosine yields DNA-protein cross-links in nucleosome core particles. J Am Chem Soc 2017;**139**:10617-10620.

Pettersen, E.F., Goddard, T.D., Huang, C.C., *et al.* UCSF Chimera—A visualization system for exploratory research and analysis. J Comput Chem 2004;**25**:1605-1612.

Punjani, A., Rubinstein, J.L., Fleet, D.J., *et al.* cryoSPARC: algorithms for rapid unsupervised cryo-EM structure determination. Nat Methods 2017;**14**:290-296.

Rosenthal, P.B., and Henderson, R. Optimal determination of particle orientation, absolute hand, and contrast loss in single-particle electron cryomicroscopy. J Mol Biol 2003;**333**:721-745.

Srinivasan, S., McGuigan, C., Andrei, G., *et al.* Bicyclic nucleoside inhibitors of varicella-zoster virus (VZV): effect of terminal unsaturation in the side-chain. NUCLEOS NUCLEOT NUCL 2001;**20**:763-766.

Studitsky, V.M., Clark, D.J., and Felsenfeld, G. A histone octamer can step around a transcribing polymerase without leaving the template. Cell 1994;**76**:371-382.

Studitsky, V.M., Clark, D.J., and Felsenfeld, G. Overcoming a nucleosomal barrier to transcription. Cell 1995;**83**:19-27.

Taguchi, H., Horikoshi, N., Arimura, Y., *et al.* A method for evaluating nucleosome stability with a protein-binding fluorescent dye. Methods 2014;**70**:119-126.

Yan, L., Wu, H., Li, X., *et al.* Structures of the ISWI–nucleosome complex reveal a conserved mechanism of chromatin remodeling. Nat Struct Mol Biol 2019;**26**:258-266.

Zhou, C., Sczepanski, J.T., and Greenberg, M.M. Histone modification via rapid cleavage of C4′-oxidized abasic sites in nucleosome core particles. J Am Chem Soc 2013;**135**:5274-5277.
